# Impacts of the Covid-19 lockdown and relevant vulnerabilities on capability well-being, mental health and social support: an Austrian survey study

**DOI:** 10.1186/s12889-021-10351-5

**Published:** 2021-02-08

**Authors:** Judit Simon, Timea M. Helter, Ross G. White, Catharina van der Boor, Agata Łaszewska

**Affiliations:** 1 Department of Health Economics, Center for Public Health, Medical University of Vienna, Kinderspitalgasse 15, 1090 Vienna, Austria; 2 Department of Psychiatry, University of Oxford, Warneford Hospital, Oxford, OX3 7JX UK; 3 Primary Care and Mental Health, Institute of Population Health, University of Liverpool, School of Psychology, Brownlow Hill, Liverpool, L69 3GB UK

**Keywords:** Covid-19, Austria, Capabilities, Well-being, Mental health, OxCAP-MH, Vulnerability, Resilience

## Abstract

**Background:**

Impacts of the Covid-19 pandemic and its public health measures go beyond physical and mental health and incorporate wider well-being impacts in terms of what people are free to do or be. We explored the impacts of the Covid-19 lockdown and relevant vulnerabilities on capability well-being, mental health and social support in Austria.

**Methods:**

Adult Austrian residents (*n* = 560) provided responses to a cross-sectional online survey about their experiences during Covid-19 lockdown (15 March-15 April 2020). Instruments measuring capabilities (OxCAP-MH), depression and anxiety (HADS), social support (MSPSS) and mental well-being (WHO-5) were used in association with six pre-defined vulnerabilities using multivariable linear regression.

**Results:**

31% of the participants reported low mental well-being and only 30% of those with a history of mental health treatment received treatment during lockdown. Past mental health treatment had a significant negative effect across all outcome measures with an associated capability well-being score reduction of − 6.54 (95%CI, − 9.26, − 3.82). Direct Covid-19 experience and being ‘at risk’ due to age and/or physical health conditions were also associated with significant capability deprivations. When adjusted for vulnerabilities, significant capability reductions were observed in association with increased levels of depression (− 1.77) and anxiety (− 1.50), and significantly higher capability levels (+ 3.75) were associated with higher levels of social support. Compared to the cohort average, individual capability impacts varied between − 9% for those reporting past mental health treatment and + 5% for those reporting one score higher on the social support scale.

**Conclusions:**

Our study is the first to assess the capability limiting aspects of lockdown and relevant vulnerabilities alongside their impacts on mental health and social support. The negative capability well-being, mental health and social support impacts of the Covid-19 lockdown were strongest for people with a history of mental health treatment. Future public health policies concerning lockdowns should pay special attention to improve social support levels in order to increase public resilience.

**Supplementary Information:**

The online version contains supplementary material available at 10.1186/s12889-021-10351-5.

## Introduction

The recently discovered coronavirus, known as severe acute respiratory syndrome coronavirus 2 (SARS-CoV-2), has spread globally within a span of a few months since December 2019 [1]. The Covid-19 disease caused by the virus was declared as a pandemic by the World Health Organisation (WHO) on 11 March 2020. Initial evidence suggested that the infection has a high effective reproduction rate with older populations and those with underlying health conditions being at high risk of severe disease and death, thereby forcing numerous countries into temporary lockdowns to limit the spread of the disease. Consequently, the Covid-19 pandemic went from a direct health emergency to a systemic crisis affecting people’s lives in multiple ways [2]. Covid-19 impacts have been unprecedented because of its evolution from a health shock to a global economic and social crisis [2].

Substantial evidence from the past studies of the impacts of Severe Acute Respiratory Syndrome, Middle East Respiratory Syndrome, and Ebola epidemics on the suffering individuals and the healthcare providers showed substantial neuropsychiatric linkage [3]. There is an increasing amount of research related to the impacts Covid-19 on people’s mental health and well-being [3–22]. Beside the direct health impact, public health emergencies may also affect individuals and communities through isolation, stigma, job insecurity, or inadequate resources for medical response [15]. These effects generate a range of emotional reactions, and can be particularly prevalent among those individuals who contract the disease, or those who are at increased risk due to their age or pre-existing medical conditions [15]. Evidence from previous pandemics shows that individuals who contracted the disease experienced fear, anxiety, emotional distress, and post-trauma stress symptoms [3]. The mental health/well-being impacts of the Covid-19 pandemic have been shown to be even more significant for those who are prone to psychological problems [6].

Impacts of the Covid19 pandemic and its public health measures go beyond physical and mental health and incorporate wider well-being impacts in terms of what people are free to do or be. Due to these complexities, the assessment of personal consequences related to well-being is challenging and may be best addressed within the conceptual framework of the capability approach introduced by Amartya Sen in the early 1980s [23]. The core focus of the capability approach is on what individuals are able to be and do in their lives, in other words, what they are capable of [23]. The capability approach provides a richer evaluative space beyond health and proposes that well-being is determined by people’s freedom to engage in forms of being and doing that are of intrinsic value to the person [23]. Beside the recently proposed use of the capability framework in the understanding of policy challenges [24], this freedom aspect can be interpreted in the narrower mental health context as both the actual capabilities of a person, for instance, good mental health, and the processes that enable them, for instance, legal regulations [25]. Not only has the Covid-19 pandemic had a profound psychological impact, but it also affects personal freedoms to engage in behaviours that are consistent with subjectively held values beyond health, for instance, visiting loved ones, engaging in recreational activities, spending time outdoors. Despite these important links, the connection between pandemics and individual capabilities have not yet been researched.

In Europe, Austria stood out as a nation that adopted aggressive and early strategies and thereby saw a smaller proportion of deaths from Covid-19 compared to some other European countries [26]. The first Covid-19 case in Austria was reported on 25 February 2020 [27]. The Austrian government issued general laws to contain the epidemic by restricting social contacts and imposing strict lockdown measures from 16 March onwards [27] most of which have been lifted gradually since 15 April.

Early studies assessing the Covid-19 pandemic, and related public health measures, impacts found significant impact on the mental health of the Austrian population. The studies found that symptoms of moderate to severe anxiety and depression have tripled in Austria, and 8–13% of the population showed severe depression and 6–11% severe anxiety symptoms [28, 29].

The capability approach embodies a range of interlinking concepts and several studies have indicated that capability outcomes are strongly associated with mental health and social outcomes [30–34]. However, despite the increasing number of studies exploring the Covid-19 impact on mental health/well-being, information is still missing on the broader capability impact of the pandemic. Hence, this study aimed to explore the impact the Covid-19 lockdown period on people’s capabilities in association with mental health/well-being and social support, especially in the case of specific vulnerable groups in Austria. Covid-19 lockdown vulnerable groups were pre-defined as: (i) being categorised as ‘at risk’ group based on age and/or pre-existing physical health conditions; (ii) self-reported mental health treatment prior to the coronavirus pandemic; (iii) direct exposure to Covid-19 (having symptoms or being tested positive); (iv) indirect exposure to Covid-19 through a family member/friend; (v) having employment status impacted by the lockdown; or (vi) being categorised as critical worker.

## Methods

### Study participants

Participants were recruited using convenience sampling, i.e. the study sample consisted of people who responded to our survey advert. The advert was distributed via multiple channels including social media platforms (including Facebook, Twitter, WhatsApp) and emails targeting a wide range of individuals and organisations (universities, non-profit organisation such as Red Cross, and local governments) throughout Austria. In order to be able to participate in the study, respondents had to be older than 18 years, have sufficient German knowledge, and be residents in Austria at the time of the Covid-19 outbreak.

### Study design and data collection

Cross-sectional data were collected via an online survey in May/June 2020, with all questions, including standardised outcome instruments, referring to the one-month lockdown period in Austria between 15 March and 15 April 2020.

The survey was developed in the SoSci online survey platform (Version 2), which is a publicly available tool and is free of charge for academic research [35]. The weblink of the survey was included in an advert, along with a QR code, that was circulated via social media platforms (including Facebook, Twitter, WhatsApp, etc.) and emails targeting a wide range of individuals and organisations throughout Austria.

Respondents who provided sociodemographic and Covid-19-related information and completed at least one standardised outcome instrument were considered for analysis. Those participants who discontinued the survey before fully completing at least one standardised outcome instrument were excluded from the analyses. The current analysis is based on the questions that were completed as part of the survey as outlined in the supplementary file (Supplementary file 1).

### Survey and instruments

The online survey consisted of the participant information and consent forms followed by a section on sociodemographics. Subsequent sections assessed people’s perceptions about the Covid-19 pandemic and the public health measures in place during the lockdown in Austria in response to the outbreak. The final part of the questionnaire consisted of four self-reported standardised and validated outcome instruments, which were used to assess capability well-being (OxCAP-MH), depression and anxiety levels (HADS), social support (MSPSS) and mental well-being (WHO-5) similar to a parallel linked survey in the UK [36]. The outcome instruments were adapted to the online survey including the adaptation of their introductory text reflecting the period of interest, i.e. the one-month lockdown period in Austria between 15 March and 15 April 2020.

The Oxford CAPabilities questionnaire-Mental Health (OxCAP-MH) instrument was developed by Simon et al. in 2013 [37]. It is specifically designed to capture different well-being dimensions within the capability framework in the area of mental health across 16 items. The OxCAP-MH is scored on a 0–100 scale, with higher scores indicating better capabilities. The OxCAP-MH has shown good psychometric properties including internal consistency (Cronbach’s alpha between 0.79 and 0.85), test-retest reliability (intra class correlation coefficient 0.80), construct validity and responsiveness in both English and German populations [33, 34]. The German version of the OxCAP-MH [38] was obtained from the authors for the study.

The Hospital Anxiety and Depression Scale (HADS) was developed by Zigmond and Snaith in 1983 [39]. The questionnaire is divided into Anxiety (HADS-A) and Depression (HADS-D) subscales both containing seven items scored on a four-point scale from zero (not present) to three (considerable). Both the HADS-A and HADS-D subscales are scored from 0 to 21, with higher scores indicating higher anxiety or depression levels. Normal, borderline and abnormal anxiety/depression scores are defined as 0–7, 8–10 and 11–21, respectively [39]. The HADS was found to perform well in assessing the presence and severity of anxiety disorders and depression in both somatic and psychiatric cases, also beyond the hospital setting, including the primary care patients and general population (mean sensitivity 0.90 and mean specificity 0.78 for a cut-off score of 8+ for HADS-A; and mean sensitivity 0.83 and mean specificity 0.79 for a cut-off score of 8+ for HADS-D) [40]. Cronbach’s alpha coefficient of internal consistency reported in several studies varied from 0.68 to 0.93 for HADS-A and from 0.67 to 0.90 for HADS-D [40]. The German translation of HADS was obtained from Hogrefe Publishing Group.

The Multidimensional Scale of Perceived Social Support (MSPSS) is a self-reported measure of subjectively assessed social support developed by Zimet et al. in 1988 [41]. The questionnaire can be divided into three subscales, each addressing a different source of support: Family, Friends, and Significant Other. Low, moderate and high support are defined as < 3, 3–5 and > 5, respectively [41]. The instrument has good internal consistency (Cronbach’s alpha 0.88) and test-retest reliability (0.85) [41]. An official German translation of MSPSS was obtained from the developer of the original English version.

The World Health Organisation-Five Well-being Index (WHO-5) is a short self-reported measure of current mental well-being introduced in 1998 by the WHO Regional Office in Europe [42]. Respondents are asked to rate how well each of the five statements about positive well-being applied to them in the given period from 5 (all of the time) to 0 (none of the time). The WHO-5 is scored 0–25, with higher scores representing higher well-being [43]. The WHO-5 has been used in multiple studies across countries and disease areas [43]. A review of 213 articles using the WHO-5 as an outcome measure confirmed that the instrument has satisfactory construct validity, responsiveness and it can be used as a screening tool for depression [43]. The German translation of the WHO-5 is available in the public domain without registration.

### Definition of vulnerabilities

In the current study, six hypothesised associations between increased levels of mental health symptoms and decreased levels of well-being were tested according to pre-defined vulnerabilities identified as relevant to Covid-19: 1) “At risk” group; 2) Past mental health treatment; 3) Direct Covid-19 experience; 4) Indirect Covid-19 experience; 5) Employment status affected by Covid-19; and 6) Critical worker. Individuals were defined as ‘at risk’ if they were aged 65 years or over and/or had a self-reported underlying physical health condition including diabetes, heart/cardiovascular disease, stroke/cerebrovascular disease, lung disease, liver disease, or cancer. Participants who reported mental health service use prior to the period of interest were categorised as ‘having past mental health treatment’. Participants with ‘direct Covid-19 experience’ were those who tested positive for Covid-19 or experienced Covid-19 symptoms, but were not tested. ‘Indirect Covid-19 experience’ was defined as having a friend and/or family member infected or knowing someone who died of Covid-19. Participants with ‘employment status affected’ were those who reported losing their job due to the pandemic or being sent to short-time working (German ‘Kurzarbeit’). Finally, participants who reported having a job categorised by the government as critical worker, e.g. healthcare staff, police officer or food supply worker, were defined as ‘critical workers’.

### Statistical analysis

Anonymous data were extracted from the online survey and checked for logical inconsistencies. Characteristics of the study cohort in comparison to the general Austrian population were presented.

Correlations between the different outcome measures were explored using Pearson’s correlations and interpreted as small < 0.3, moderate 0.3–0.49, or large ≥0.50 [44]. In order to explore the impacts of the Covid-19 lockdown and relevant pre-defined vulnerabilities on capabilities, mental health/well-being and social support, multivariable linear regression analyses were conducted using the OxCAP-MH, HADS-D, HADS-A, MSPSS and WHO-5 scores as dependent variables and six binary variables that defined vulnerable groups as independent variables. Analyses were adjusted for age, gender, having children, education level and initial employment status.

The potential impact of current depression, anxiety and social support on capabilities was investigated separately in a multivariable regression with OxCAP-MH capability score as the dependent variable and HADS-D, HADS-A, MSPSS scores as independent variables, adjusted for sociodemographic characteristics (age, gender, having children, education level and initial employment status) and the six relevant vulnerabilities as described above. Significance level of *p* < 0.05 was considered in all analyses. Analyses were conducted on complete cases in STATA v.15.1 [45].

## Results

### Participant characteristics

**Fig. 1:**
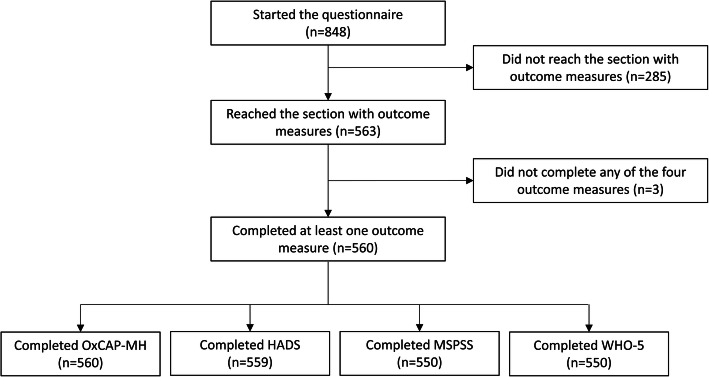
Survey flowchart

Of the 848 persons who accessed the survey, 560 respondents (74.1% female, mean age *M* = 40.22 years, *SD* = 11.60) completed it and were included in the analyses (Fig. 1). The average time needed to complete the survey was 17 min.

The majority of participants were Austrian citizens (87%) and employed at the beginning of the Covid-19 lockdown (73%). More than half of the survey participants (56%) had children, 52% were married or had a registered partnership. Data relating to the sociodemographic characteristics of the sample compared to official Austrian population statistics, with respect to age, gender, distribution of population across federal states [46], migration background [47], education level [48], and employment status [49], are shown in Table 1.

**Table 1:** Characteristics of the survey cohort

	Covid-19 Study ***n*** = 560		Austrian population
	n	%	%
Gender			
Female	416	74%	51%
Male	143	26%	49%
Diverse	1	0%	
Missing	0	0%	
Age			
18–29	97	17%	18%
30–49	319	57%	34%
50–64	124	22%	26%
65–79	13	2%	23%
Missing	7	1%	
Federal state			
Burgenland	12	2%	3%
Carinthia	41	7%	6%
Lower Austria	109	20%	19%
Upper Austria	66	12%	17%
Salzburg	25	5%	6%
Styria	63	11%	14%
Tyrol	24	4%	9%
Vorarlberg	5	1%	4%
Vienna	215	38%	21%
Missing	0	0%	
Migration background			
EU-Members before 2004/EWR/Switzerland	33	6%	3%
EU-Members joined 2004 and after	14	3%	5%
Former Yugoslavia (not-EU), Turkey	7	1%	3%
Other countries	12	2%	5%
Austrian roots	489	87%	84%
Missing	5	1%	
Education			
Primary education	13	2%	26%
Vocational school for apprentices	68	12%	32%
Intermediate technical and vocational school	45	8%	14%
General secondary education and vocational colleges	132	24%	15%
Higher education	302	54%	13%
Missing	0	0%	
Employment status			
Housekeeping	28	5%	2%
Student	37	7%	4%
Employed	410	73%	64%
Self-employed	37	7%	9%
Unemployed	16	3%	2%
Retired	25	4%	19%
Missing	7	1%	
Family status			
Single	204	36%	
Married or registered partnership, living together	266	48%	
Married or registered partnership, living separately	20	4%	
Widowed	6	1%	
Divorced	46	8%	
Missing	18	3%	
Children			
Yes	311	56%	
No	245	44%	
Missing	4	1%	
Categorised as ‘at risk’ group^a^			
Yes	72	13%	
No	486	87%	
Missing	2	0%	
Received mental health treatment before the pandemic			
Yes	97	17%	
No	456	81%	
Missing	7	1%	
Received mental health treatment during the pandemic lockdown			
Yes	34	6%	
No	518	93%	
Missing	7	1%	
Direct Covid-19 experience	39	7%	
Tested positive for Covid-19	7	1%	
Experienced symptoms of Covid-19, not tested	32	6%	
Missing	0	0%	
Indirect Covid-19 experience^b^	110	20%	
Close friend tested positive for Covid-19	46	9%	
Family member tested positive for Covid-19	32	6%	
Knew someone who died of Covid-19	44	8%	
Missing	0	0%	
Employment status affected by Covid-19	84	15%	
Fired from job	15	3%	
Short-time working	69	12%	
Missing	6	1%	
Critical worker			
Yes	214	38%	
No	330	59%	
Missing	16	3%	
OxCAP-MH score (mean, SD)	74.10	12.30	
Missing	0	0%	
HADS-D (mean, SD)	4.72	4.09	
HADS depression score normal (0–7)	436	78%	
HADS depression score borderline (8–10)	62	11%	
HADS depression score abnormal (11–21)	61	11%	
Missing	1	0%	
HADS-A (mean, SD)	6.26	4.19	
HADS anxiety score normal (0–7)	362	65%	
HADS anxiety score borderline (8–10)	108	19%	
HADS anxiety score abnormal (11–21)	89	16%	
Missing	1	0%	
MSPSS score (mean, SD)	5.64	1.23	
MSPSS high support (5.1–7)	416	74%	
MSPSS moderate support (3–5)	112	20%	
MSPSS low support (0–2.99)	22	4%	
Missing	10	2%	
WHO-5 score (mean, SD)	15.10	4.80	
Missing	10	2%	

^a^ Participants were categorised as ‘at risk’ group if they were aged 65 or more, and/or they self-reported at least one of the listed diseases: heart/cardiovascular disease, stroke/cerebrovascular disease, lung disease (e.g. asthma, cystic fibrosis, COPD), liver disease (e.g. hepatitis), cancer;

^b^ Respondents included in “direct Covid-19 experience” variable were excluded from this group

### Vulnerabilities

A total of 13% of the respondents (*N* = 72) were categorised as belonging to the ‘at risk’ group based on age and/or co-existing physical health conditions. While 17% of the participants (*N* = 97) reported that they received treatment for mental disorders before the period of interest, only 6% of the participants (*N* = 34) reported receiving mental health treatment during the pandemic. Overall, only 30% of those with a mental health service use history (*N* = 29) reported receiving treatment also during the lockdown.

A total of 1% of participants (*N* = 7) had been diagnosed with Covid-19, another 6% (*N* = 32) of the participants experienced Covid-19-like symptoms without being tested, and 20% of the respondents (*N* = 110) had indirect Covid-19 experience through an infected friend and/or family member, or knew someone who died of Covid-19. Employment status was affected for 15% (*N* = 84) of participants (job terminated: 3%, *N* = 15; short-term work: 12%, *N* = 69), and 38% of the respondents (*N* = 214) reported having a job categorised as ‘critical worker’ (Table 1).

The level of missing values for the standardised outcome instruments was low with a maximum of ten observations missing (1.8%) for MSPSS and WHO-5. The mean OxCAP-MH score was 74.10 (*SD* = 12.30). The mean WHO-5 score was 15.10 (*SD* = 4.80) with 31% (*N* = 174) of the respondents reporting a score below 13 indicating low mental well-being [42]. The mean scores on HADS-A and HADS-D subscales were 6.26 (*SD* = 4.19) and 4.72 (*SD* = 4.09), respectively, indicating that respondents on average reported higher levels of anxiety than depression symptoms. A total of 74% of participants (*N* = 416) reached the threshold of > 5 for high social support on the MPSS scale. Average scores for the MSPSS subscales were 5.41 for family support, 5.53 for support from friends and 5.96 for support from significant others.

### Correlations between capability well-being, mental health/well-being and social support outcomes

Capability well-being (OxCAP-MH) was significantly strongly/moderately associated with all other outcome measures, the strongest correlation being with depression (HADS-D: *r* (557) = −.64, *p* < .01; HADS-A: *r* (557) = −.56, *p* < .01; WHO-5: *r* (448) = .58, *p* < .01; MSPSS: *r* (448) = .42, *p* < .01). In terms of social support, capabilities and depression had the same strength of correlations, but of opposite directions. (Table 2).

**Table 2:** Correlations between capability well-being, mental health/well-being and social support outcomes

	OxCAP-MH	HADS-D	HADS-A	MSPSS	WHO-5
OxCAP-MH	1				
HADS-D	−0.64***	1			
HADS-A	−0.56***	0.75***	1		
MSPSS	0.42***	−0.42***	−0.30***	1	
WHO-5	0.58***	−0.70***	−0.67***	0.34***	1

Note: * *p* < 0.05, ** *p* < 0.01, *** *p* < 0.001; *OxCAP-MH* Oxford CAPabilities questionnaire-Mental Health; *HADS-D* Hospital Anxiety and Depression Scale-Depression subscale, *HADS-A* Hospital Anxiety and Depression Scale-Anxiety subscale, *MSPSS* Multidimensional Scale of Perceived Social Support, *WHO-5* World Health Organisation-Five Well-being Index

### Outcome associations with different types of vulnerability

Outcome associations with different types of vulnerabilities adjusted for sociodemographics are shown in Table 3. Past mental health treatment had a significant negative effect across all outcome measures with an associated capability well-being score reduction of − 6.54 (*b* = − 6.54, *t* (502) = − 4.73, *p* < .01), while direct Covid-19 experience had the second most detrimental impact with an associated capability well-being score reduction of − 4.58 (*b* = − 4.58, *t* (502) = − 2.27, *p* = 0.02). Capabilities were similarly negatively affected also for those who belonged to the category ‘at risk’ (*b* = − 4.45, *t* (502) = − 2.70, *p* < .01). These correspond to capability deprivations of − 9% and − 6%, respectively, when compared to the average capability level of the study cohort.

**Table 3:** Associations between capability well-being, depression, anxiety, social support, mental well-being and different types of vulnerabilities

Vulnerabilities	OxCAP-MH score		HADS-D score		HADS-A score		MSPSS score		WHO-5 score	
	ß (SE)	95% CI	ß (SE)	95% CI	ß (SE)	95% CI	ß (SE)	95% CI	ß (SE)	95% CI
At risk group	−4.45** (1.65)	−7.68, − 1.21	0.29 (0.56)	−0.80, 1.40	0.82 (0.57)	−0.30, 1.93	0.03 (0.17)	−0.31, 0.38	− 0.80 (0.66)	−2.09, 0.50
Past mental health treatment	− 6.54*** (1.38)	−9.26, − 3.82	1.97*** (0.47)	1.05, 2.90	2.09*** (0.48)	1.16, 3.03	−0.44** (0.15)	−0.74, − 0.15	−1.61** (0.56)	− 2.71, − 0.50
Direct Covid-19 experience	− 4.58* (2.01)	−8.54, − 0.62	1.23 (0.69)	−0.12, 2.58	1.77* (0.69)	0.40, 3.13	−0.09 (0.22)	−0.49, 0.35	−1.59* (0.82)	−3.20, 0.00
Indirect Covid-19 experience	−0.11 (1.29)	−2.65, 2.43	0.25 (0.44)	−0.61, 1.12	0.97* (0.45)	0.09, 1.85	−0.31* (0.14)	−0.59, − 0.04	0.04 (0.53)	−0.99, 1.08
Employment status affected	−2.31 (1.55)	−5.36, 0.74	0.80 (0.53)	−0.24, 1.84	0.81 (0.53)	−0.24, 1.90	0.01 (0.16)	−0.33, 0.31	−1.20 (0.62)	−2.43, 0.03
Critical worker	0.08 (1.12)	−2.13, 2.30	−0.33 (0.38)	−1.08, 0.43	−0.32 (0.39)	−1.09, 0.44	− 0.07 (0.12)	−0.30, 0.17	− 0.49 (0.45)	−1.38, 0.40
Constant	68.90*** (4.83)	59.41, 78.38	3.08 (1.65)	−1.15, 6.32	6.44*** (1.66)	3.17, 9.71	6.11*** (0.51)	5.11, 7.12	16.35*** (1.93)	12.55, 20.15

Note: Standard errors (SE) in parentheses * *p* < 0.05, ** *p* < 0.01, *** *p* < 0.001; Statistically significant coefficients (*p* < 0.05) in bold; Analyses adjusted for age, gender, migration status, education level, having children, employment status; *OxCAP-MH* Oxford CAPabilities questionnaire-Mental Health, *HADS-D* Hospital Anxiety and Depression Scale-Depression subscale, *HADS-A* Hospital Anxiety and Depression Scale-Anxiety subscale, *MSPSS* Multidimensional Scale of Perceived Social Support, *WHO-5* World Health Organisation-Five Well-being Index

Having employment status affected by the pandemic produced consistently lower capability and mental well-being scores as well as higher depression and anxiety scores, but these associations did not reach statistical significance. We did not observe any significant impacts for the category ‘critical worker’ either.

### Associations between capability well-being and current depression, anxiety and social support levels

Additional associations between current levels of depression and anxiety as well as social support with capability well-being were investigated in a separate multivariable regression analysis adjusted for all vulnerabilities and sociodemographics (Table 4). Current levels of depression and anxiety separately showed a capability score reduction of − 1.77 (*b* = − 1.77, *t* (500) = − 16.89, *p* < .01) and − 1.50 (*b* = − 1.50, *t* (500) = − 13.52, *p* < .01), respectively, per one point difference in the relevant HADS scores. Social support on the other hand proved to be a major capability resilience factor. One point score improvement on the MSPSS scale was associated with an improvement of + 3.75 (*b* = 3.75, *t* (491) = 9.60, *p* < .01) in the capability scores.

**Table 4:** Associations between capability well-being and current depression (HADS-D), anxiety (HADS-A) and social support (MSPSS) levels

	OxCAP-MH	
	ß (SE)	95% CI
HADS-D	−1.77 (0.10)***	−1.97, − 1.56
HADS-A	− 1.50 (0.11)***	−1.72, − 1.28
MSPSS	3.75 (0.39)***	2.98, 4.51

Note: * *p* < 0.05, ** *p* < 0.01, *** *p* < 0.001; Analyses adjusted for age, gender, migration status, education level, having children, employment status, at risk group for Covid-19, past mental health treatment, direct Covid-19 experience, indirect Covid-19 experience, employment status affected by Covid-19, critical worker; *OxCAP-MH* Oxford CAPabilities questionnaire-Mental Health, *HADS-D* Hospital Anxiety and Depression Scale-Depression subscale, *HADS-A* Hospital Anxiety and Depression Scale-Anxiety subscale, *MSPSS* Multidimensional Scale of Perceived Social Support, *WHO-5* World Health Organisation-Five Well-being Index

## Discussion

This is the first study to assess the impact of the Covid-19 lockdown and relevant vulnerabilities on capabilities well-being, mental health and social support and their associations as observed in Austria.

Our findings that Covid-19 direct experience is associated with intensified anxiety symptoms, lower mental well-being and lower capabilities are in line with other recent studies exploring the impact of the Covid-19 pandemic on mental health and well-being in Austria [28, 29, 50–52]. Our study showed that participants who reported mental health treatment before the Covid-19 pandemic reported worse outcomes on all measures, including the OxCAP-MH, HADS-D, HADS-A, MSPSS and WHO-5. However, only the OxCAP-MH capability questionnaire showed a significant negative impact for participants categorised as belonging to the ‘at risk’ group. It should be noted that it is likely that the Covid-19 lockdown restrictions accentuated levels of distress experienced by those with existing physical health conditions. This association has not been captured by any other outcome measure, suggesting an increased sensitivity of the OxCAP-MH in comparison to the other scales used in this study and confirming the advantage of its broader measurement scope when assessing the well-being impact of a pandemic and related public health measures. The study also confirmed that the capability approach, which provides an indication of people’s freedom to engage in forms of being and doing that are of intrinsic value to the person, has direct relevance to situations/policies that inherently limit personal freedoms, i.e. public health emergencies.

The vulnerabilities referred to in this study as ‘employment status affected’ by Covid-19 or being a ‘critical worker’ were not significantly associated with any of the outcomes. Besides the issue of sample size, this may also reflect the Austrian government’s employment support policy implemented in the early stages of the pandemic including the introduction of the short-term working scheme to help retain jobs [53, 54].

When considering the average capability well-being score observed in our cohort, the relative impact of different vulnerabilities and other factors on capability levels were estimated between − 9% for those reporting past mental health treatment vs. + 5% for reporting one score higher on the social support scale. In future analyses, the outcome scores obtained in this study could also be compared to scores observed in studies prior to the Covid-19 pandemic to further asses the overall impact of this public health emergency and lockdown on the well-being of the Austrian population. Previous studies using the WHO-5 instrument found that 26–27% of the Austrian sample reported scores corresponding to low mental well-being [55, 56]. This is lower than the 31% of respondents who were identified as having low mental well-being (WHO-5 score below 13) in our study. Furthermore, 19% of the participants in this study had borderline and 16% ‘abnormal’ anxiety levels according to HADS-A scoring system, somewhat higher than the levels reported in earlier Austrian studies [57–60]. These results seem to be confirmative of the expected negative impacts of the Covid-19 pandemic, including those of the lockdown, on mental well-being including increased levels of anxiety and stress. Previous studies using the MSPSS scale in Austrian populations reported comparable scores, indicating relatively high social support [61, 62].

In addition to providing an indication of the Covid-19 and lockdown impacts on vulnerable groups, this study also highlighted the interactions between capability well-being levels and current mental health that indicate a strong negative impact of current depression and anxiety. On the other hand, social support was shown as a major capability resilience factor. Future (public health) policies should take the strong associations between capabilities and current mental health and social support levels directly into consideration to minimise the negative long-term health, social and economic issues related to future public health emergencies.

Furthermore, our results suggest that amongst all investigated vulnerabilities, people with past mental health treatment represent the most vulnerable group. A recent study from Austria found that the number of people treated with psychotherapy during lockdown (personal, phone or virtual contacts) decreased by one-third [63]. In our study, the proportion of people receiving mental health treatment during lockdown in comparison to the period before the pandemic was 6% vs. 17%, respectively. We found evidence of the continuation of treatment between the two periods for only 30% of those participants who received mental health treatment prior to the pandemic. Even under the most conservative assumptions, these results suggest a substantial level of under-utilisation of mental health services (due to whatever causes) during the lockdown period. For future strategic healthcare planning during next waves of the pandemic, policy makers and health and social care providers need to be aware of the exceptional vulnerability of this group and efforts should be focused on maintaining continuity in mental health service provision. Digital e-health treatment options provide potential solution to assure this continuity of treatment whilst simultaneously protecting the health of the service-users and professionals [64, 65].

The results of this research also have major implications for government departments, social care services and community-based support initiatives in planning how best to support the population during future pandemics, and in terms of the special attention needed for those with pre-existing mental health service use. Findings also provide crucial evidence for policy makers and members of the public by indicating how important and protective social support networks can be in mitigating the mental health and (capability) well-being impacts of public health emergencies through increased resilience. The latter finding goes beyond the health sector with relevant implications also for the education sector when considering decisions about university openings and necessary support networks for students. Future research should explore whether the observed impacts on capabilities, mental health and social support levels remain, worsen or diminish (via adjustment) as the pandemic continues and how they develop in the long-term after the public health situation is resolved.

Furthermore, strategies that can help to alleviate the negative impacts of the Covid-19 lockdown in the Austrian population should be identified. Priority should be given to assuring the continuity of mental health services, as well as identifying new cases of mental disorders, mental distress and anxiety that might arise due to lockdowns. This will require the capacity of mental health support services to be increased, policy initiatives to be communicated in a clear and transparent way so that anxiety is reduced among the population, and the introduction of work arrangements that allow for home-schooling for both parents (reducing the burden posed on mothers) that prevent loss of household income [66]. Moreover, governments should implement economic measures and reinforce essential health, social and education services to identify population needs, reduce inequalities in health and protect most vulnerable citizens including people with pre-existing conditions, elderly, migrant population, children and those with lower socio-economic status [67].

The main limitation of our study is that the participants completed the survey retrospectively about 1 month after the lockdown (mid-May 2020). This time-lag may have introduced some recall bias considering the self-reported outcome measures. Since data were collected at the time when the number of new Covid-19 cases were relatively low and the Austrian epidemic curve has flattened, we assume that the presented estimates are more conservative and optimistic than if the survey questions would have been completed directly during the lockdown. Moreover, since the analysis is based on one measurement point, the study allows no causal conclusions. Our study is also prone to limitations of online survey; results are based fully on self-reporting with the potential to reporting bias [68] and some groups (females, younger ages, higher educated), were over-represented in the survey sample compared to the general population [69, 70]. The survey on the other hand achieved satisfactory representation in terms of more than half of the Austrian provinces, migration background and employment status.

## Conclusions

This research contributes to the understanding of the impact that pandemics and nationwide responses to pandemics can have on mental health and broader capability well-beings in light of their major policy relevance. Furthermore, the study confirms that the OxCAP-MH capability measure is a valid and relevant tool to understand the impacts of the Covid-19 pandemic and related public health measures, which due to the negative externalities of any infectious disease inherently limit individual freedoms to some extent. Future research is planned to compare cultural aspects of lockdown experiences across countries and explore long-term mental health/well-being impacts from the perspective of the capability approach.

## Supplementary Information

**Additional file 1.**

## Data Availability

The datasets generated during the current study and the study protocol have been released in a scientific data repository and can be accessed under the link: 10.5281/zenodo.4271534.

## Abbreviations

HADS: The Hospital Anxiety and Depression Scale
MSPSS: The Multidimensional Scale of Perceived Social Support
OxCAP-MH: The Oxford CAPabilities questionnaire-Mental Health
WHO: World Health Organisation
WHO-5: The World Health Organisation-Five Well-being Index
