# Supplementary material for: Impacts of the Covid-19 lockdown and relevant vulnerabilities on capability well-being, mental health and social support: an Austrian survey study
Source: BMC Public Health. 2021 Feb 8;21:314. doi: 10.1186/s12889-021-10351-5 (PMC7868863; doi:10.1186/s12889-021-10351-5)
Supplement: Supplementary file 1 — Additional file 1. [file 12889_2021_10351_MOESM1_ESM.docx]

**Supplementary file 1**

**Impacts of the Covid-19 lockdown and relevant vulnerabilities on capability well-being, mental health and social support: An Austrian survey study**

Judit Simon^1,2^, Timea M. Helter^1^, Ross G. White^3^, Catharina van der Boor^3^, Agata Łaszewska^1^

^1^ Department of Health Economics, Center for Public Health, Medical University of Vienna, Kinderspitalgasse 15, 1090 Vienna, Austria

^2^ Department of Psychiatry, University of Oxford, Warneford Hospital, Oxford OX3 7JX, UK

^3^ Primary Care and Mental Health, Institute of Population Health, University of Liverpool, School of Psychology, Brownlow Hill, Liverpool, L69 3GB, UK

**Corresponding Author**

Judit Simon, Department of Health Economics, Center for Public Health, Medical University of Vienna, Kinderspitalgasse 15, 1090 Vienna, Austria; Phone: +43 1 40160 34841; E-Mail: [judit.simon@meduniwien.ac.at](mailto:judit.simon@meduniwien.ac.at)

**List of questionnaire sections:**

1. Demographic section
2. COVID-19 questions
3. OxCAP-MH
4. Hospital Anxiety and Depression Scale
5. Multidimensional Scale of Perceived Social Support
6. WHO-5 Well-being measure
7. **Demographic section**

While answering the questionnaire, please always think about the 4 weeks between mid-March and mid-April 2020 when the stay-home restrictions in Austria were at their peak.

1. Gender

Male

Female

Diverse

1. What is your age? (Years from 18 to 90 in numbers). If you prefer not to give this information then please leave it blank.
2. Do you have a migration background?

No

EU countries prior to 2004 / EEA / Switzerland

EU accession countries from 2004

Former Yugoslavia (non-EU), Turkey

Other countries

1. What is your highest educational achievement?

Primary school

Apprenticeship with vocational school

Technical or commercial school

“Matura”

Degree from a university, (technical) college

Any other higher degree following “Matura”

5. In which federal state was your main residence?

Burgenland

Carinthia

Lower Austria

Upper Austria

Salzburg

Styria

Tyrol

Vorarlberg

Vienna

6. What was the reason for your stay in Austria?

I am an Austrian citizen

I am an asylum seeker or a refugee

I work in Austria

I am here on vacation

I am studying here

Other reason (please specify)

I prefer not to provide this information

7. What was your marital status in mid-March 2020?

Single

Married or registered partnership, living together

Married or registered partnership, separated

Widowed

Divorced

I prefer not to provide this information

1. Do you have any children?

Yes

No

I prefer not to provide this information

1. If yes, how many?
2. What was your employment status in mid-March 2020?

I was not employed (e.g. household)

Student

Employed

Self-employed

Civil servant

Unemployed

Retired

I prefer not to provide this information

1. If you are currently employed, what type of employment do you do?

Business, consulting and management

Accountancy, banking and finance

Charity and voluntary work

Creative arts and design

Energy and utilities

Engineering and manufacturing

Environment and agriculture

Healthcare

Hospitality and events management

Information technology

Law

Law enforcement and security

Leisure, sport and tourism

Marketing, advertising and PR

Media and internet

Property and construction

Public services and administration

Recruitment and HR

Retail

Sales

Science and pharmaceuticals

Social care

University lecturer or Researcher

Teacher

Transport and logistics

I prefer not to provide this information

1. Have you been fired due to COVID-19?

Yes

No

I prefer not to provide this information

1. Have you been sent to short-term working (“Kurzarbeit”) due to COVID-19?

Yes

No

I prefer not to provide this information

1. Did you receive treatment or support for a mental health problem during COVID-19 lockdown?

No

Yes

I prefer not to provide this information

1. If yes, please state which mental health problem:

Acquired brain injury or neurological disorder (i.e. dementia, brain damage, delirium)

Mental and behavioural disorders due to psychoactive substance use (i.e. alcohol, or drugs)

Psychosis (including schizophrenia, schizotypal or delusional disorders)

Mood disorders (i.e. depression or bipolar affective disorder,)

Neurotic, stress-related and somatoform disorders (i.e. anxiety disorders, obsessive compulsive disorder)

Behavioural syndromes associated with physiological disturbances and physical factors (i.e. eating disorders, sleeping disorders)

Disorders of adult personality and behaviour (i.e. specific personality disorders, gender identity disorders)

Disorders of psychological development (i.e. Asperger's syndrome, specific reading disorders)

I prefer not to provide this information

1. Did you receive treatment or support for a mental health problem prior to COVID-19?

No

Yes

I prefer not to provide this information

1. If yes, please state which mental health problem:

Acquired brain injury or neurological disorder (i.e. dementia, brain damage, delirium)

Mental and behavioural disorders due to psychoactive substance use (i.e. alcohol, or drugs)

Psychosis (including schizophrenia, schizotypal or delusional disorders)

Mood disorders (i.e. depression or bipolar affective disorder,)

Neurotic, stress-related and somatoform disorders (i.e. anxiety disorders, obsessive compulsive disorder)

Behavioural syndromes associated with physiological disturbances and physical factors (i.e. eating disorders, sleeping disorders)

Disorders of adult personality and behaviour (i.e. specific personality disorders, gender identity disorders)

Disorders of psychological development (i.e. Asperger's syndrome, specific reading disorders)

I prefer not to provide this information

1. Are you currently receiving treatment for (yes/no/prefer not to say):

Diabetes

Heart/cardiovascular disease

Stroke/cerebrovascular disease

Lung disease (e.g. Asthma, Cystic Fibrosis, COPD)

Liver disease (e.g. Hepatitis)

Cancer

1. **COVID-19 Questions**
2. I have tested positive for COVID-19

Yes

No

1. I have experienced the symptoms of COVID-19 including persistent cough and high body temperature (above 37.8 degrees Celsius) since the outbreak in Austria began but was not tested.

Yes

No

1. I have a close friend who has tested positive for COVID-19

Yes

No

1. I have one or more immediate family members who have tested positive for COVID-19

Yes

No

1. I have known someone who died as a consequence of COVID-19

Yes

No

1. I work in a job that is categorised by the government as an essential/key role

Yes

No

I prefer not to provide this information

1. **OxCAP-MH [1]**


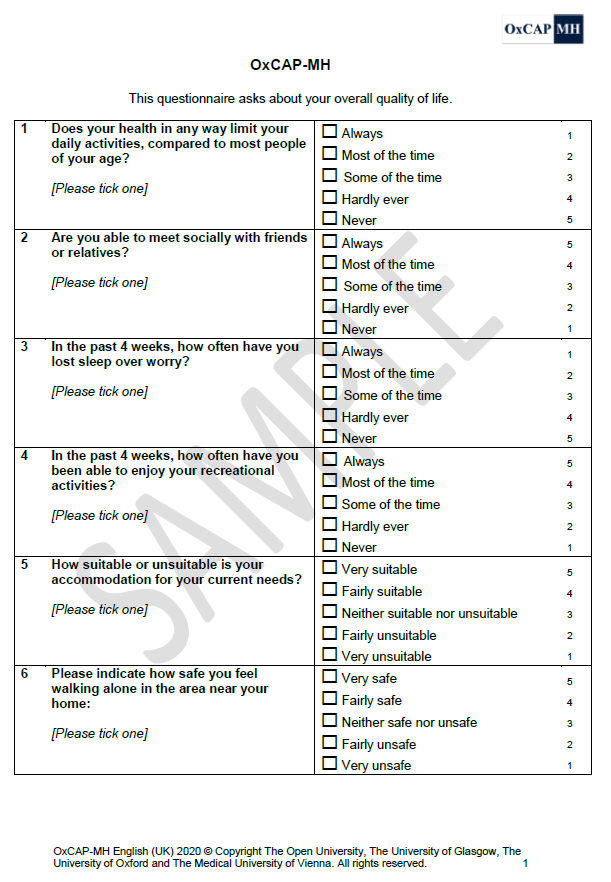


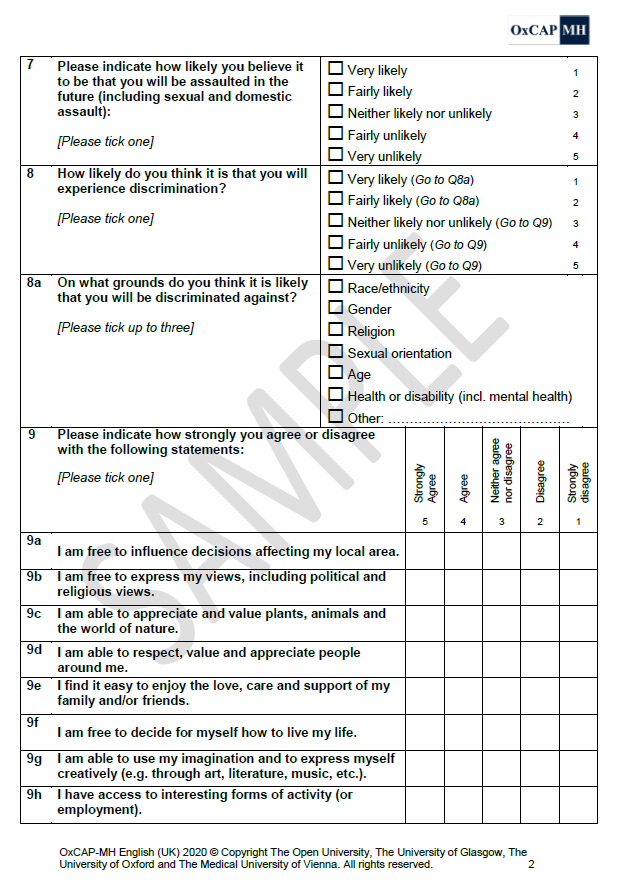


1. **Hospital Anxiety and Depression Scale (HADS) [2]***

*Author’s note: A request to the authors of the questionnaire to reproduction is in progress. To access the full version of the questionnaire, contact:

GL Assessment Ltd.
1st Floor Vantage London
Great West Road
London TW8 9AG
United Kingdom
Email: [permissions@gl-assessment.co.uk](mailto:permissions@gl-assessment.co.uk)
Website: [www.gl-assessment.co.uk](http://www.gl-assessment.co.uk)

1. **Multidimensional Scale of Perceived Social Support [3]**


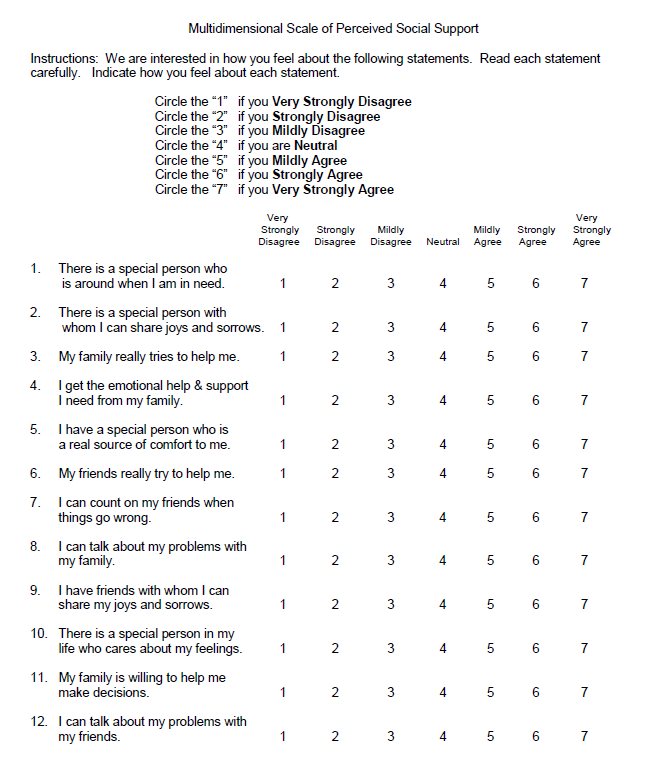


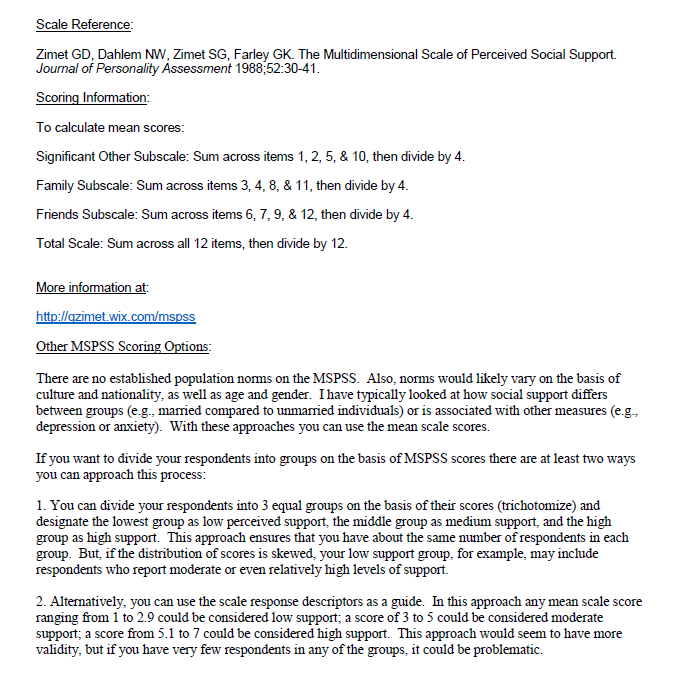


1. **WHO (Five) Well-Being Index [4]**


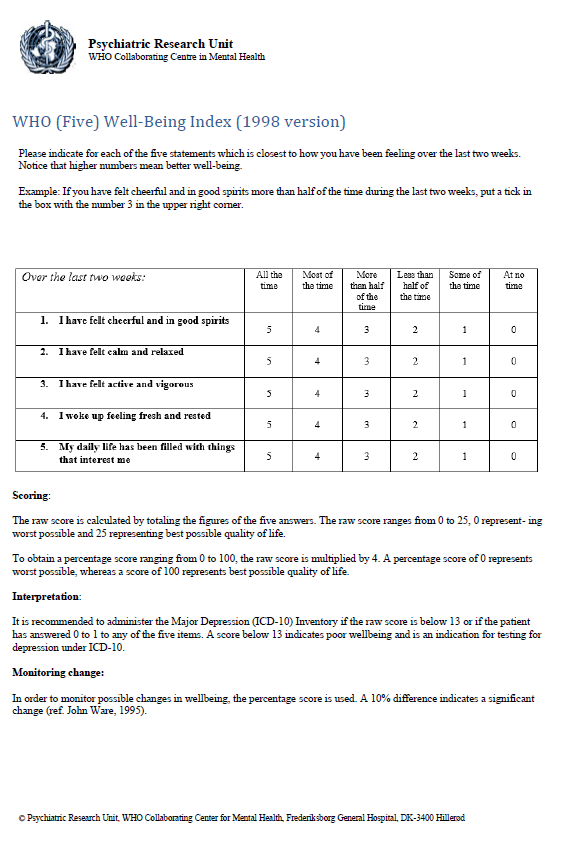


**References**

1 Simon J, Anand P, Gray A, Rugkåsa J, Yeeles K, Burns T. Operationalising the capability approach for outcome measurement in mental health research. Soc Sci Med. 2013;98:187-96

2 Snaith RP, Zigmond AS. The hospital anxiety and depression scale. Br Med J (Clin Res Ed). 1986;292(6516):344

3 WHO. Well-being Measures in Primary Health Care/The Depcare Project. Report on a WHO Meeting. WHO Regional Office for Europe. Copenhagen 1998

4 Zimet GD, Dahlem NW, Zimet SG, Farley GK. The Multidimensional Scale of Perceived Social Support. J Pers Assess. 1988;52(1):30-41
